# Government livelihood project - free influenza vaccination for the older population in China: the example of Zhejiang Province

**DOI:** 10.3389/fpubh.2025.1571499

**Published:** 2025-05-19

**Authors:** Xiaoxiao Wang, Wei Jiang, Zhao Yu, Weiping Jiang, Xiaowei Zhu, Zhen Wang, Jimin Sun, Hangjie Zhang

**Affiliations:** ^1^Department of Prevention and Control of Infectious Disease, Zhejiang Provincial Center for Disease Control and Prevention, Hangzhou, China; ^2^Key Laboratory of Vaccine, Prevention and Control of Infectious Disease of Zhejiang Province, Zhejiang Provincial Center for Disease Control and Prevention, Hangzhou, China; ^3^Haiyan County Center for Disease Control and Prevention, Jiaxing, China

**Keywords:** influenza vaccine, livelihood projects, older population, strategy, Zhejiang

## Abstract

Influenza vaccination is an effective measure to prevent and control influenza, and countries worldwide consider older people a key recommended population for influenza vaccination. From 2020, Zhejiang Province has provided free influenza vaccination to anyone aged ≥70 years as part of its livelihood projects. In certain counties, the program is also offered to those aged ≥60 years. This measure has made influenza vaccination more convenient and has advanced the strategy for acceptance of influenza vaccines and improved readiness for a pandemic. Furthermore, it serves as an example for other Chinese provinces looking to implement free influenza vaccination programs.

## Introduction

Influenza is an acute respiratory infectious disease caused by the influenza virus. Influenza poses a serious health risk to the population, and vaccination is the most cost-effective intervention to prevent influenza and its complications (1). Many countries have implemented annual seasonal influenza vaccination, especially among older adults, who are usually at a higher risk of influenza infection, severe complications, and death than other groups (2). Influenza vaccination is not included in the national immunization program of China. A national cross-sectional survey showed that the overall influenza vaccination rate was extremely low (3.16 and 2.47%) in 2021 and 2022 (3). Additionally, only a few developed regions in China have influenza vaccination policies. The vaccination rate is 32.94 to 45.71% for those aged ≥60 years, far lower than the World Health Organization recommendation of 75% and lower than the vaccination rate in developed countries (3–6).

The Chinese government places a high value on immunizing the older population against influenza. In “Healthy China Action (2019–2030),” the “14th Five-Year Plan,” and other plans and documents, the government has highlighted the need to ensure that the supply of influenza vaccines is adequately managed. Additionally, according to China’s Technical Guidelines for Influenza Vaccination (2023–2024), influenza vaccination should be prioritized for high-risk groups, such as patients with certain chronic diseases and people aged ≥60 years (7). To reduce the harm owing to influenza infection and strengthen the prevention and control of influenza in the older population, Zhejiang Province has included influenza vaccination for people aged ≥70 years in its livelihood projects and has implemented a voluntary free vaccination policy from 2020.

Here, we conducted a descriptive policy evaluation using administrative health records and vaccination data from Haiyan County, Zhejiang Province, collected between 2019 and 2023, along with interview data for case analysis.

### Project of free influenza vaccination

The Zhejiang provincial government implements a number of useful initiatives each year to enhance residents’ quality of life, with a primary focus on housing, work, health care, and education. The free influenza vaccination for older residents aged ≥70 years as a part of Zhejiang’s livelihood projects has achieved the highest ranking in both performance evaluation and satisfaction assessment. According to an on-site survey, the satisfaction rate for immunization tops 99%. By 2023, the program covered all districts and counties in the province for those aged ≥70 years, with an additional 17 districts and 15 counties covering individuals aged ≥60 and ≥65 years, benefiting more than 1.9 million older adults. A total of 1,459/1,501 (97.2%) of immunization and vaccination clinics and over 13,000 medical staff have been involved in project implementation.

Haiyan County, which belongs to Jiaxing City, is located in northern Zhejiang Province, with a jurisdiction of four streets and five towns and a residential population of 471,000 people. Haiyan County has been implementing its free influenza vaccination project from 2019, earlier than the provincial project and extended of the vaccinated population to those aged ≥60 years. The annual vaccination rate was 61.0% (95%CI 57.7–64.2%) from 2019 to 2023 (Table 1), for the older people ≥70 years the vaccination rate was 56.6% (95% CI 51.0–62.2%) from 2020 to 2023, higher than province’s average vaccination rate of 36.4%.

**Table 1:** Free influenza vaccination for the older population in Haiyan County.

Year	Vaccination population *n* (%)		Vaccination notices^*^	Promotional posters/banners^†^	Funding^§^	Vaccination units
	≥60 years	≥70 years				
2019	66,031 (63.6)	27,985 (65.7)	110,000	2,000	4.39	14
2020	65,959 (63.6)	28,549 (52.4)	120,000	300	3.12	13
2021	64,531 (61.0)	30,056 (55.1)	120,000	420	2.96	11
2022	62,646 (58.4)	30,196 (58.6)	120,000	450	2.90	11
2023	66,278 (58.3)	32,874 (60.3)	120,000	420	2.90	17

^*^“Letter to the older people,” “Vaccination notices.”

^†^Vaccination service, procurement of influenza vaccine, cold-chain equipment, publicity expenses including printed materials, animated short film, television and video advertising, and other. Unit means million.

^§^Posters used in 2019, banners used in 2020–2023 years.

## Implementation and experience in Haiyan County

### Effective organization and management

Haiyan County follows a government-led, health administration department-managed, town (street)-administrative village (community)-village team leader three-tier grid organization and mobilization mode. The county propaganda department, finance bureau, public security bureau, medical security bureau, market supervision administration, health administration, and the towns (streets) departments are among the members of the lead group that the Haiyan County government formed for its free influenza vaccination project. The “Implementation Plan of Haiyan County Free Influenza Vaccination Project for the Older People over 60 Years of Age” is formulated and issued, along with a clear delegation of responsibilities among relevant government institutions to ensure smooth project execution every year.

**Figure 1:**
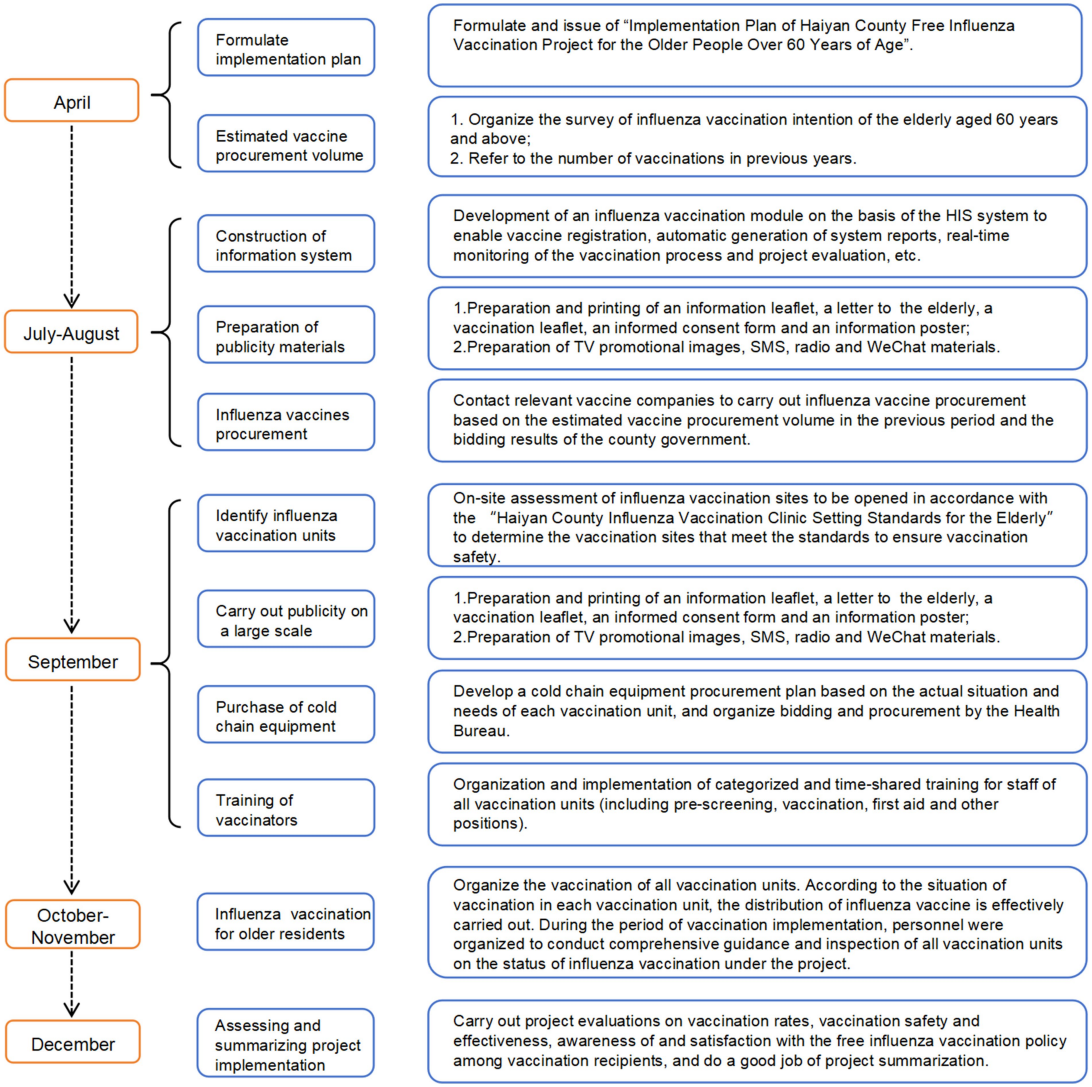
Timeline of free influenza vaccination project in Haiyan County.

Health administration department is responsible for overseeing, organizing and coordinating the administration of influenza vaccines, and ensuring that immunization procedures are conducted in a safe and well-organized manner. Haiyan County Center for Disease Control and Prevention (Haiyan CDC) is responsible for formulating implementation plan, influenza vaccines estimation and procurement, preparation of publicity materials; conducting special training for the staff of vaccination units, providing detailed explanations about influenza and influenza vaccines, the pre-screening process, the content of the work, specific operational steps and key points, and the procedure of influenza vaccination; a daily influenza vaccination reporting system is in place to keep track of vaccination progress, and specialized vehicles are used to ensure that vaccines are distributed safely and uniformly to all vaccination unites on time; investigating, and resolving suspected adverse events following immunization (AEFI). Vaccination units include community health centers (“town health centers”) and temporary vaccination sites are responsible for immunization services in accordance with regulations (Figure 1).

### Adequate publicity and mobilization

During the publicity and mobilization process, giving full play to the functions of grass-roots organizations and grids. Traditional and new media, online and offline, are used in combination, and the core knowledge of influenza prevention and control is widely publicized in a variety of time slots and forms.

Using widely disseminated and mobilized mass media, such as television, the Internet, microblogging, and other outlets, actively distribute publicity materials (posters, radio spots, SMS messages, and other diverse publicity) to publicize and popularize the risks associated with influenza, the importance of vaccination, the free vaccination policy, and the vaccination appointment time. Village and committees actively distribute leaflets, put up posters, issue broadcasts, send text messages, and use other various forms of media to promote the free influenza vaccination program. Posters were placed on health education bulletin boards in the fixed health education positions such as hospitals, towns, and villages, and images and cards were particularly printed. Group inoculations are administered at regular intervals and in regular dosages, and villages (communities) are notified in batches.

A total of 120,000 copies of the “Letter to older Friends” and “Vaccination Notification Form,” 85,000 copies of the “Vaccination Informed Consent Form” and 420 sets of publicity posters were printed in Hanyan every year (Table 1). At the same time, brief promotional movies were meticulously created to expand the reach of the publicity.

### Appointments and convenient vaccination conditions

Through information technology, the online “Zheli Office” APP and the offline contracted services of family doctors have been combined to provide convenient services for the public, including scheduling vaccination appointments, extending the vaccination cycle, performing time-sharing vaccinations, extending the daily service hours, and opening special sessions on weekends and holidays. To maximize the quality of services and improve public convenience, make full use of the COVID-19 vaccination hatch and mobile vaccination, among other initiatives, to deliver services flexibly, open green channels, and kindly accompany the entire process. Nearly all community health centers, also known as “town health centers,” offer free influenza vaccinations, and additional temporary vaccination locations are being developed in response to the requirements of the older adult population. Sufficient funding, between 2.90 and 4.39 million RMB year, is provided to support the project’s successful completion.

### Consultation and supervision

A service hotline has been established to receive complaints and inquiries from the public, respond to questions from the public, promptly address the demands of the older population, and carefully and scientifically answer questions. In addition, government have stepped up their monitoring of public opinion within their borders and have paid particular attention to calls and visits from the public as well as online public opinion.

A special supervisory team is responsible for the implementation of vaccination at the vaccination point and providing on-site supervision, timely detection of problems, and the implementation of corrections, to ensure that the entire vaccination process follows standard implementation procedures.

## Discussion

Influenza poses a greater potential health hazard for older than younger populations. Influenza vaccination is the most effective measure for preventing and controlling the disease, substantially reducing the risk of influenza, influenza-related complications, and even death, with important health and economic benefits for most older people, particularly those with chronic underlying diseases (8).

Vaccine hesitancy, a delay in accepting or refusing vaccination despite the availability of vaccination services, has grown in recent years (9). Despite enacting complementary strategies, including improving vaccination services, promoting vaccination facilitation, and strengthening policy promotion, influenza vaccine coverage has been unsatisfactory (10). Due to lack of knowledge about protection of vaccines against influenza, worry about adverse effects of vaccines, cost of vaccines, and inconvenience in obtaining vaccines, older individuals are mostly neglecting to get influenza vaccination.

Free influenza vaccination livelihood projects have benefited people aged ≥70 years at the provincial, municipal, and county levels in China. Localities have also supplemented provincial projects through livelihood projects at the municipal and county levels, with free influenza vaccination for the population aged ≥60 years, which has comprehensively increased the influenza vaccine coverage rate among older residents. This project is conducive to creating an immune barrier in high-risk older adult people and has great public health importance for reducing the incidence of influenza-related illnesses, visits to hospital, hospitalizations, and serious illnesses in this population.

With smooth implementation of the livelihood project for 4 consecutive years, rich practical experience has been accumulated at all levels. This project features an advanced design and scientific planning; multiple rounds of publicity and training as well as multi-level and on-site supervision and guidance focusing on risk points within the entire process; scientific and standardized organization and implementation; monitoring of AEFI and public opinion in multiple locations; and responding to demands and concerns of older groups scientifically, carefully, and in a timely manner. These efforts have effectively improved the risk prevention capability of the project and ensured the smooth organization and implementation of the project without any major safety and public opinion incidents.

Through implementation of the project, a number of unites continue to use information technology, COVID-19 vaccination pods, mobile vaccination, flexible forms of service, open and green channels, and provide caring accompaniment to enhance convenience for the public and optimize the quality of service. The willingness to receive influenza vaccination among older residents of municipalities in Zhejiang Province has increased each year. The improvement and optimization of vaccination service satisfaction has also increased and has effectively enhanced the health of this population. It has also enhanced the recognition of the government’s practical efforts to improve people’s livelihoods, sense of achievement, and well-being by safeguarding their health.

Despite the significant improvement in vaccination rates, this study has several limitations. First, while the increase in vaccination rates among the older adult is notable, the study does not fully elucidate the key factors driving behavioral changes in this population. Future research should employ a mixed-methods approach, combining quantitative and qualitative methods, to explore how intersectoral collaboration-such as the role of family doctor contract services-can reduce vaccination barriers and enhance the effectiveness of vaccination programs. Second, this study did not assess the long-term financial sustainability of the vaccination program. As the program is considered for scaling up, it is crucial to explore alternative funding mechanisms, such as the incorporation of medical insurance fund contributions or the introduction of market-based financing models, to ensure the program’s fiscal viability over time. Third, while the vaccination model demonstrated in this study is more easily replicable in resource-intensive regions, such as developed counties in eastern China, its implementation in rural areas of central and western China may face significant challenges. Specifically, addressing the human resource shortages in these regions will be critical to ensuring the model’s adaptability and scalability in less-resourced settings.

In conclusion, the Government’s Livelihood Project-Free Influenza Vaccination for the Older Population in Zhejiang Province has comprehensively increased the influenza vaccine coverage rate among older residents in the province at the provincial, municipal and county levels. This project’s successful promotion helps us to increase promotion of free influenza vaccination programs for province-wide older population aged ≥60 years and other high-risk groups.

## Data Availability

The raw data supporting the conclusions of this article will be made available by the authors, without undue reservation.
