# Correction: Government livelihood project - free influenza vaccination for the older population in China: the example of Zhejiang Province

**DOI:** 10.3389/fpubh.2025.1655995

**Published:** 2025-07-21

**Authors:** Xiaoxiao Wang, Wei Jiang, Zhao Yu, Weiping Jiang, Xiaowei Zhu, Zhen Wang, Jimin Sun, Hangjie Zhang

**Affiliations:** ^1^Department of Prevention and Control of Infectious Disease, Zhejiang Provincial Center for Disease Control and Prevention, Hangzhou, China; ^2^Key Laboratory of Vaccine, Prevention and Control of Infectious Disease of Zhejiang Province, Zhejiang Provincial Center for Disease Control and Prevention, Hangzhou, China; ^3^Haiyan County Center for Disease Control and Prevention, Jiaxing, China

**Keywords:** influenza vaccine, livelihood projects, older population, strategy, Zhejiang

There was a mistake in Table 1 as published. Due to policy restrictions, the AEFI (Adverse Events Following Immunization) data for the Government Livelihood Project cannot be disclosed. We have decided to remove this data due to considerations related to public health information management and policy compliance. The corrected Table 1 appears below.

**Table 1:** Free influenza vaccination for the older population in Haiyan County.

**Year**	**Vaccination population;** ***n*** **(%)**		Vaccination notices^*^	**Promotional posters/banners^†^**	**Funding^§^**	**Vaccination units**
	**≥60 years**	**≥70 years**				
2019	66,031 (63.6)	27,985 (65.7)	110,000	2,000	4.39	14
2020	65,959 (63.6)	28,549 (52.4)	120,000	300	3.12	13
2021	64,531 (61.0)	30,056 (55.1)	120,000	420	2.96	11
2022	62,646 (58.4)	30,196 (58.6)	120,000	450	2.90	11
2023	66,278 (58.3)	32,874 (60.3)	120,000	420	2.90	17

^*^“Letter to the older people,” “Vaccination Notices.”

^†^Vaccination service, procurement of influenza vaccine, cold-chain equipment, publicity expenses including printed materials, animated short film, television and video advertising, and other. Unit means million.

^§^Posters used in 2019, banners used in 2020–2023 years.

In the published article, there was a mistake in the text. The year of the data described in the sentence “By 2024, the program covered all districts and counties in the province for those aged ≥70 years, with an additional 17 districts and 15 counties covering individuals aged ≥60 and ≥65 years, benefiting more than 1.9 million older adults.” was wrong. The correct year of the data is 2023.

A correction has been made to the section **Introduction**, *Project of Free Influenza Vaccination*, Paragraph 1:

“By 2023, the program covered all districts and counties in the province for those aged ≥70 years, with an additional 17 districts and 15 counties covering individuals aged ≥60 and ≥65 years, benefiting more than 1.9 million older adults.”

In the published article, the Ethics statement was not included. The Ethics statement should be:

“This study involving human participants was exempt from ethical review and approval by the Ethics Committee of the Zhejiang Provincial Center for Disease Control and Prevention, and informed consent from [patients/participants or their legal guardians/next of kin] was not required in accordance with the committee's regulations.”

